# IoMT Architecture for Fully Automated Point-of-Care Molecular Diagnostic Device

**DOI:** 10.3390/s25144426

**Published:** 2025-07-16

**Authors:** Min-Gin Kim, Byeong-Heon Kil, Mun-Ho Ryu, Jong-Dae Kim

**Affiliations:** 1 Thermo Fisher Scientific, South San Francisco, CA 90080, USA; hellingford@gmail.com; 2 School of Software, Hallym University, Chuncheon-si 24252, Republic of Korea; zsewa0@hallym.ac.kr; 3 Division of Biomedical Engineering, Jeonbuk National University, Jeonju 54896, Republic of Korea; 4 Research Center of Healthcare & Welfare Instrument for the Aged, Jeonbuk National University, Jeonju 54896, Republic of Korea

**Keywords:** Internet of Medical Things, point-of-care diagnostics, molecular diagnostics, real-time PCR, cloud-based architecture, WebSockets, infectious disease detection

## Abstract

The Internet of Medical Things (IoMT) is revolutionizing healthcare by integrating smart diagnostic devices with cloud computing and real-time data analytics. The emergence of infectious diseases, including COVID-19, underscores the need for rapid and decentralized diagnostics to facilitate early intervention. Traditional centralized laboratory testing introduces delays, limiting timely medical responses. While point-of-care molecular diagnostic (POC-MD) systems offer an alternative, challenges remain in cost, accessibility, and network inefficiencies. This study proposes an IoMT-based architecture for fully automated POC-MD devices, leveraging WebSockets for optimized communication, enhancing microfluidic cartridge efficiency, and integrating a hardware-based emulator for real-time validation. The system incorporates DNA extraction and real-time polymerase chain reaction functionalities into modular, networked components, improving flexibility and scalability. Although the system itself has not yet undergone clinical validation, it builds upon the core cartridge and detection architecture of a previously validated cartridge-based platform for *Chlamydia trachomatis* and *Neisseria gonorrhoeae* (CT/NG). These pathogens were selected due to their global prevalence, high asymptomatic transmission rates, and clinical importance in reproductive health. In a previous clinical study involving 510 patient specimens, the system demonstrated high concordance with a commercial assay with limits of detection below 10 copies/μL, supporting the feasibility of this architecture for point-of-care molecular diagnostics. By addressing existing limitations, this system establishes a new standard for next-generation diagnostics, ensuring rapid, reliable, and accessible disease detection.

## 1. Introduction

The Internet of Medical Things (IoMT) is revolutionizing the healthcare landscape by integrating smart medical devices, cloud computing, and real-time data analytics to enhance patient care and diagnostic accuracy [1,2,3,4]. Recent reviews have also highlighted the potential of IoMT-based point-of-care diagnostic platforms to address current limitations in decentralized testing environments [5]. IoMT enables remote monitoring, real-time disease detection, and seamless data exchange between healthcare providers and diagnostic systems. This interconnected approach is particularly critical in point-of-care molecular diagnostics, where rapid and decentralized testing is essential for timely disease management.

Infectious diseases caused by various pathogens, such as bacteria, viruses, and parasites, pose a significant threat to public health due to their high transmissibility. Major infectious diseases, including respiratory illnesses (e.g., influenza, MERS, and COVID-19) and sexually transmitted infections (e.g., *Chlamydia trachomatis* and *Neisseria gonorrhoeae*), have significantly affected public health and, in the case of respiratory epidemics, the global economy [6,7,8,9,10,11].

The COVID-19 pandemic, in particular, has demonstrated the devastating consequences of highly infectious diseases, not only in terms of morbidity and mortality but also through economic downturns, disruptions in education, and adverse effects on mental health [10,12]. As a result, mitigating the spread of infectious diseases has become a crucial public health priority, necessitating effective control measures such as patient isolation. Rapid and accurate diagnosis is essential for implementing timely isolation protocols and guiding appropriate treatment strategies. The ability to quickly detect and analyze pathogens plays a critical role in controlling outbreaks and informing public health responses [10,12,13]. For instance, in the case of influenza, viral replication occurs within 72 h post-infection, making early antiviral administration (within 48 h of symptom onset) crucial for optimal treatment outcomes. Similarly, in conditions like severe sepsis, prompt pathogen identification is necessary for early antibiotic intervention, emphasizing the need for rapid point-of-care (POC) diagnostic methods.

Traditional diagnostic approaches rely on centralized laboratory testing, where patient samples must be transported to major hospitals or specialized laboratories. This process is time-consuming, delaying disease control efforts and reducing the effectiveness of early interventions. In contrast, point-of-care molecular diagnostic (POC-MD) methods offer a promising solution by enabling rapid and decentralized testing, facilitating early detection, infection control, and improved healthcare monitoring [14]. The World Health Organization (WHO) initially established the ‘ASSURED’ criteria—Affordable, Sensitive, Specific, User-friendly, Rapid and Robust, Equipment-free, and Deliverable—as the gold standard for ideal POC diagnostic devices [5]. This has since evolved into the ‘REASSURED’ criteria, which incorporates real-time connectivity and ease of specimen collection to reflect the requirements of modern, networked diagnostics [15]. The system proposed in this study was developed with these updated criteria in mind. To address key REASSURED elements in practical field use, the system incorporates a pre-filled reagent cartridge to reduce per-test cost (affordability), supports tunable thermal cycling protocols for robust operation across sample conditions (robustness), and allows direct sample input without nucleic acid extraction (equipment-free). These design choices aim to enhance deployability in low-resource environments.

Among the existing POC diagnostic techniques, rapid antigen tests and molecular diagnostics are widely utilized [12]. Rapid antigen tests, which were frequently employed during the COVID-19 pandemic, provide quick and convenient testing; however, their accuracy limitations result in unreliable outcomes [16]. Moreover, real-time polymerase chain reaction (real-time PCR) is the most widely recommended molecular diagnostic method for COVID-19 detection due to its high sensitivity and specificity. However, traditional real-time PCR systems are expensive and require skilled personnel, making them less accessible for point-of-care applications [8,17,18,19,20]. Addressing these challenges requires the development of compact, cost-effective, and user-friendly real-time PCR systems that integrate automated sample processing, from nucleic acid extraction to detection, within a single embedded system [21,22,23].

Embedded systems play a critical role in the development and operation of diagnostic devices. However, maintaining and modifying such systems often require significant time, cost, and resources. The adoption of open-source software and hardware offers an effective strategy for accelerating development, facilitating maintenance, and leveraging community-driven problem-solving [24,25,26,27,28].

Advancements in information technology have also influenced the evolution of user interfaces (UIs) in embedded systems. While traditional graphical user interfaces (GUIs) have been implemented using built-in displays or PC applications, the increasing integration of web technologies is shifting GUI development towards web-based interfaces that offer seamless compatibility across multiple devices, including smartphones [29,30,31,32].

Furthermore, the connectivity of diagnostic systems has evolved from wired interfaces (e.g., USB) to wireless technologies such as Bluetooth and Wi-Fi, enhancing flexibility in device operation. The incorporation of cloud-based solutions enables remote data collection and system maintenance [33]. However, traditional software architectures often struggle with device management in Industrial Internet of Things (IIoT) environments. A modular and distributed system architecture is necessary for efficient device lifecycle management, real-time operation, and seamless integration of networked components [32,34,35,36].

This study proposes an advanced system architecture for a fully automated POC-MD device, incorporating recent IT advancements. Unlike conventional hierarchical architectures, our approach employs a distributed system design with interconnected functionalities, enabling remote monitoring and control via networked communication. The system leverages an optimized application programming interface (API) protocol to ensure efficient real-time operation and integrates a web-based GUI for enhanced user interaction [37,38]. Additionally, the core functions of DNA extraction and real-time PCR are structured as independent modules, allowing for autonomous operation and improved diagnostic performance. Furthermore, the core functions of DNA extraction and real-time PCR are modularized as independently operable units, each accessible via a dedicated Web interface. This structure facilitates separate testing and debugging of each biochemical step, enhancing development efficiency, reliability, and maintainability.

Our previous study introduced a cloud-based software architecture for POC-MD systems [39], demonstrating feasibility but revealing limitations in network latency, system synchronization, and user interaction efficiency. To address these challenges, this study aims to enhance the cloud-based architecture by optimizing network communication through WebSockets, thereby reducing latency. WebSocket-based communication has been shown to significantly reduce latency and overhead compared to REST or HTTP polling, particularly in real-time data transmission scenarios [40]. Additionally, the microfluidic cartridge operation is refined to improve sample processing efficiency, ensuring more reliable and consistent diagnostic outcomes. To validate real-time system performance, a hardware-based emulator—improved from our previous implementation—was constructed to replicate system behavior under real-time conditions. This platform enabled system-level testing of the software architecture, communication protocols, and user interface responsiveness prior to clinical deployment. Furthermore, user interface responsiveness is enhanced to provide seamless real-time monitoring, ensuring a smooth and intuitive experience for medical professionals using the diagnostic system.

To address limitations in previous IoMT-based POC-MD systems—including high network latency, inefficient sample processing, and limited system validation—we propose a cloud-connected diagnostic platform with several key enhancements. These include the adoption of WebSocket communication to reduce latency, optimized control of microfluidic cartridge operations, and the use of a hardware-based emulator for software verification before deployment. By integrating these improvements, the proposed system aims to set a new standard for next-generation POC-MD methods, ensuring rapid, reliable, and accessible disease detection. While the proposed IoMT-based version has not yet undergone clinical testing, it builds upon a cartridge and detection architecture that was clinically validated in a commercial diagnostic device for the simultaneous detection of *Chlamydia trachomatis* and *Neisseria gonorrhoeae*.

## 2. Materials and Methods

### 2.1. Overview of Previous Hardware Architecture

**Figure 1:**
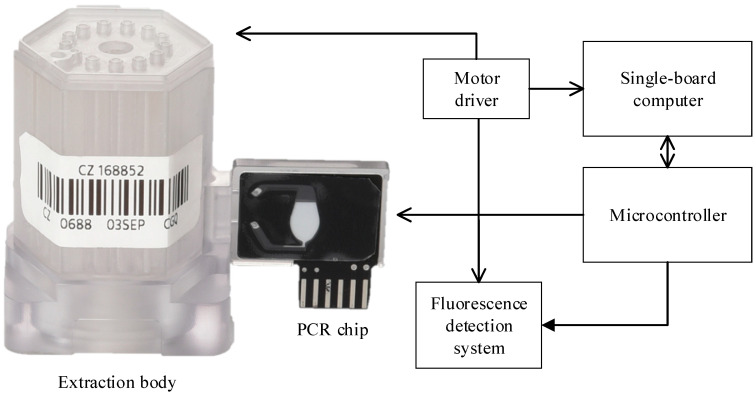
Diagram of the proposed system. The target microfluidic cartridge consists of an extraction body and PCR chip (left). A block diagram of the control system is shown on the right.

The previous system architecture consists of a microfluidic cartridge and a control system designed for automated DNA extraction and real-time PCR analysis. It was designed to process minimally invasive samples, such as swabs and urine, with minimal user intervention—fulfilling the REASSURED criterion of ease of specimen collection. Figure 1 presents the block diagram of the target microfluidic cartridge and control system [39]. The additional illustrations of the system components that support Figure 1 are provided in the Supplementary Materials. Supplementary Figure S1 presents the detailed structure of the microfluidic cartridge, including the extraction body and PCR chip used in both the previous and current systems. A centrally positioned rotation valve at the bottom of the cartridge enables selective fluid transfer between chambers, providing physical isolation during each step and preventing cross-contamination. Supplementary Figure S2 shows a more detailed system-level block diagram that complements Figure 1 by illustrating the hardware modules and their interconnections within the diagnostic platform. Supplementary Figure S3 provides a photograph of the implemented system, with key components such as the rotation stage, syringe, magnet, and optic module labeled for clarity.

Supplementary Figure S4 displays the main page of the web-based user interface developed for the proposed system that combines the physical architecture with the software interface. This user interface shows real-time amplification curves, temperature monitoring, protocol execution status, and final diagnostic results, demonstrating how users interact with the system during diagnostic workflows.

The microfluidic cartridge includes an extraction body for DNA isolation and a PCR chip for amplification and fluorescence detection. The extraction body contains multiple independent chambers, each holding a sample, magnetic beads for DNA extraction, and reagents. A centrally located syringe barrel, along with a rotation valve positioned at the bottom, facilitates the movement of fluids between chambers and the PCR chip.

After DNA extraction, the isolated DNA is transferred to the reaction chamber of the PCR chip. This top part of the reaction chamber is covered with a transparent polycarbonate layer for fluorescence detection. A thermistor embedded in the printed circuit board monitors the chamber temperature, while a heater pattern is responsible for temperature control. The primary system controller is a single-board computer (SBC), which controls multiple motors via dedicated motor drivers, including the rotation valve motor for reagent flow control, syringe motor for fluid movement, magnet motor for DNA extraction, and position sensors for precise actuation. Temperature control of the PCR chip is managed by a microcontroller unit (MCU, PIC18F4553, Microchip Technology Inc., Chandler, AZ, USA). The MCU controls the thermistor, heater pattern, and cooling fan to achieve precise thermal cycling during PCR. Additional implementation details are described in our previous study [36,39].

**Figure 2:**
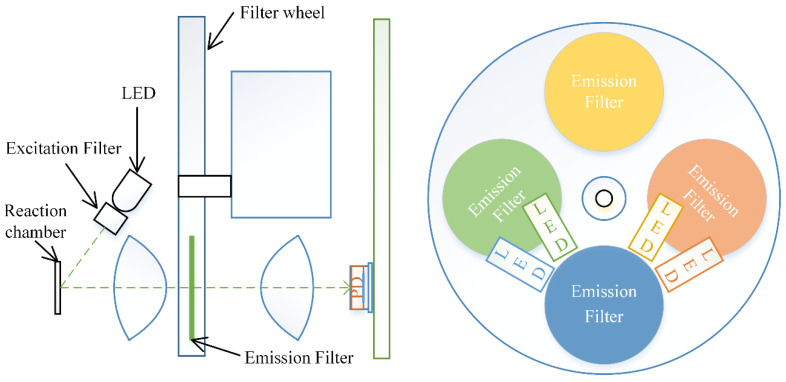
Schematic of the fluorescence detection system.

Figure 2 illustrates the fluorescence detection system, which employs four pairs of light-excitation filters and emitting diodes (LEDs) positioned at a 45° angle near the PCR chip. The PCR chip is illuminated by the emitted LED light passing through the excitation filter and is captured through an emission filter mounted on a custom-designed rotating filter wheel. A set of four matched excitation and emission filter pairs was sourced from Chroma Technology Corporation (Bellows Falls, VT, USA) for multi-channel fluorescence detection. The excitation LEDs and photodiodes were purchased from Digi-Key Electronics (Thief River Falls, MN, USA). This 4-channel configuration enables simultaneous detection of up to three distinct fluorescence-labeled targets plus an internal control, supporting triplex pathogen detection in a single reaction chamber.

**Figure 3:**
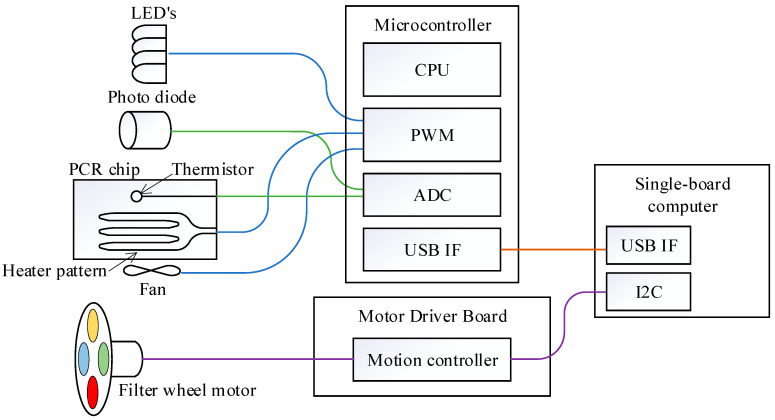
A diagram of the SBC and microcontroller connection for fluorescence detection.

Figure 3 provides an overview of the integration of a sensor–actuator with the MCU. The photodiode and thermistor are connected to the analog-to-digital converter (ADC) of the MCU, while LEDs, heater pattern, and cooling fan are controlled via pulse-width modulation (PWM). The MCU communicates with the SBC through a USB interface, ensuring efficient data exchange. To maintain consistent fluorescence measurements, the MCU directly regulates LED activation and temperature control. A proportional-integral-derivative (PID) control algorithm is implemented to precisely regulate temperature by driving the heater pattern based on real-time resistance values obtained from the thermistor.

Furthermore, motion controllers connected to the SBC’s I2C interface regulate the DNA extraction components, including the rotation valve motor, syringe motor, and magnet servo motor, as well as the fluorescence detection components, such as the filter wheel motor and position sensors.

### 2.2. Software Architecture

The previous system utilized a distributed software architecture, segmenting DNA extraction, qPCR amplification, and fluorescence detection into independent modules for flexible network-based control [39]. However, it exhibited performance limitations, including high-latency representational state transfer (REST) API communication affecting real-time operations, suboptimal microfluidic control, and a basic UI framework that impaired user experience. To address these challenges, the transition from REST API to WebSockets was implemented, enabling real-time, bidirectional communication, reducing network overhead, and improving execution speed. Additionally, an emulator-based system validation approach was integrated to simulate device behavior, allowing for comprehensive testing before deployment. A widely adopted reactive UI framework was selected to facilitate future iteration cycles between UI and engineering teams during product deployment and system refinement while also enabling further enhanced real-time visualization of diagnostic results. These improvements collectively optimized software efficiency, responsiveness, and overall system performance in automated molecular diagnostics.

**Figure 4:**
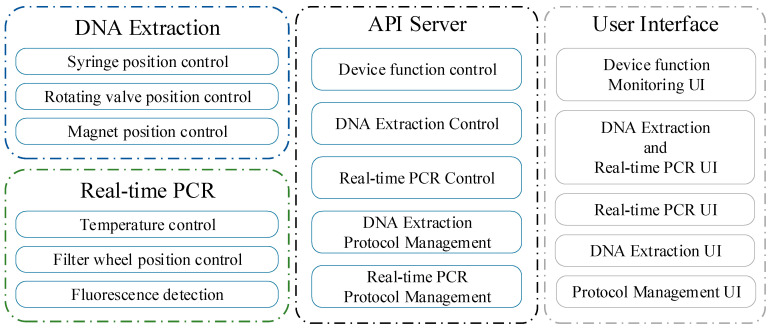
The functional architecture of the proposed software system.

Figure 4 outlines the functional architecture of the software system, categorizing functionalities into DNA extraction control, which regulates syringe position, rotation valve position, and magnetic bead movement, and real-time PCR control, which manages PCR chip temperature, filter wheel position, LED excitation, and fluorescence detection. A centralized API server facilitates DNA extraction and real-time PCR execution while providing device state monitoring and UI integration. The UI supports device monitoring, automated molecular diagnostic operation, DNA extraction, real-time PCR execution, and protocol management.

**Figure 5:**
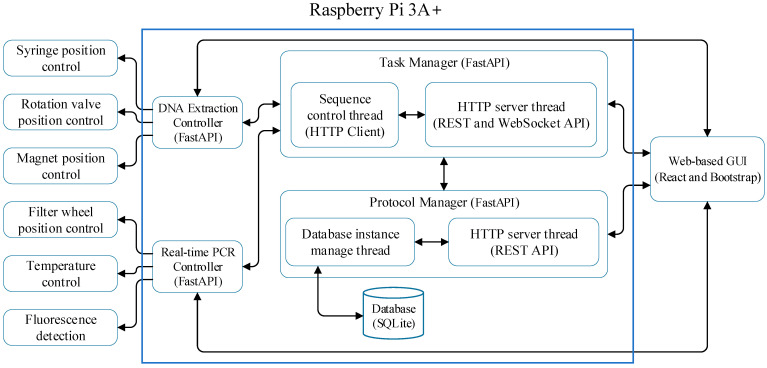
The interaction diagram of the overall software system.

For inter-process communication, the system primarily utilizes HTTP APIs. However, frequent API requests for concurrent UI updates generate excessive network traffic. To mitigate this, WebSockets are employed for high-frequency updates. Figure 5 represents the interaction diagram of the complete software system, which consists of a task manager, protocol manager, DNA extraction controller, and real-time PCR controller. Each module operates as an independent process, communicating via HTTP APIs or WebSocket APIs, ensuring efficient data exchange and modularity.

## 3. Experimental Validation and Results

The proposed system was implemented and evaluated to verify its effectiveness. The hardware system was built around a Raspberry Pi 3A+ (Raspberry Pi Foundation, Cambridge, UK) as the main controller, selected for its compact size, low power consumption, fast boot time, adequate performance, and cost-effectiveness. Its large developer community also provides a robust support ecosystem, which facilitates debugging and long-term maintenance. A PIC18F4553 microcontroller (Microchip Technology Inc., Chandler, AZ, USA) was used for thermal control, leveraging our extensive prior experience and firmware know-how for precise PCR temperature cycling. Its built-in USB HID interface also simplified communication with the SBC. The motion control system utilized L6470 stepper motor drivers (STMircoelectronics, Geneva, Switzerland), connected through I2C using SC18IS602B bridge modules (NXP Semiconductors, Eindhoven, The Netherlands). The motor controller board managed the rotation valve motor, syringe motor, and filter wheel motor, integrating AMT203 rotary encoders (Same Sky, Lake Oswego, OR, USA) for precise positioning, while a servo motor controlled the magnet motor via PWM.

The software system, which integrates modular task and protocol managers with real-time controllers, was developed using Python’s FastAPI (Python 3.11.4) for HTTP and WebSocket APIs, with SQLite for database management, as illustrated in Figure 5. The system supports WebSocket-based communication for real-time updates between the GUI and controller. This satisfies the REASSURED criterion of real-time connectivity. The web-based GUI was built using React and Bootstrap CSS and hosted on a Raspberry Pi web server. The system incorporated multicast Domain Name System (mDNS) to allow users to connect via hostname, derived from the MCU’s serial number, simplifying device discovery and access.

### 3.1. Task Manager and Protocol Manager

The task manager comprises an HTTP server thread handling API requests and a task sequence control thread managing DNA extraction and real-time PCR protocols. API requests for execution and termination are processed via HTTP and WebSocket, enabling concurrent system updates on the web-based GUI. The protocol manager, responsible for database interactions, supports operations such as retrieval, creation, modification, and deletion of protocols.

### 3.2. DNA Extraction Controller

**Figure 6:**
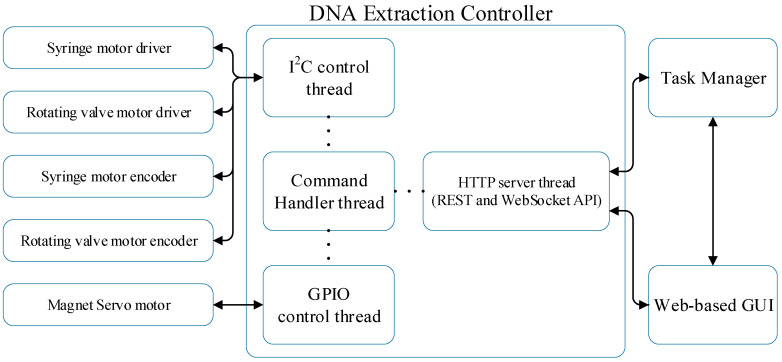
Interaction diagram of the DNA extraction controller.

The interaction diagram of the DNA extraction controller is shown in Figure 6**.** It consists of an HTTP server thread, a command handler thread for parsing high-level commands into executable actions, an I2C control thread for stepper motor management, and a GPIO control thread for magnet servo motor control. The controller communicates with the task manager via WebSocket API for periodic status updates.

To ensure efficient and synchronized hardware control, the DNA extraction controller adopts a multi-threaded architecture. Commands are classified into two types: instance commands, which require immediate execution, and queued commands, which involve time-consuming hardware operations. The command handler thread executes instance commands directly—for example, status checks or parameter updates—without delay. In contrast, commands that involve stepper motors or servo actuation are placed into queues managed by the I2C and GPIO control threads, respectively. These control threads asynchronously process queued commands in order, allowing precise and non-blocking control of the hardware components.

The DNA extraction system includes refined high-level commands described in our previous study [39], incorporating enhanced positioning and monitoring functions. Additional commands include precise motor positioning and jogging, along with AMT203-based homing capabilities. Motion controller parameters can also be adjusted for optimized DNA extraction.

Figure 6 provides an overview of the controller’s architecture and its integration with the task manager. A more detailed breakdown of the internal threading model and command handling structure is available in Supplementary Figure S5.

### 3.3. Real-Time PCR Controller

**Figure 7:**
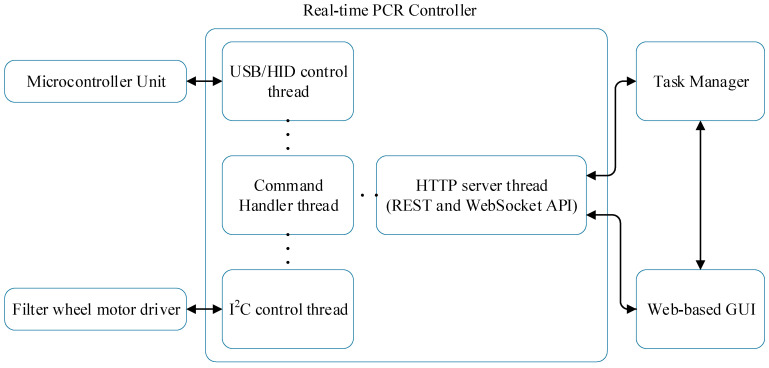
Interaction diagram of the real-time PCR controller.

Figure 7 depicts the interaction diagram of the real-time PCR controller, which comprises an HTTP server thread, a command handler thread, a USB/HID control thread for MCU communication, and an I2C control thread for filter wheel control. The PCR chip temperature is regulated using the USB/HID thread, controlling heater patterns, thermistors, and fans. Fluorescence detection is performed by managing LEDs and photodiodes through USB/HID, while filter wheels are controlled via I2C.

**Figure 8:**
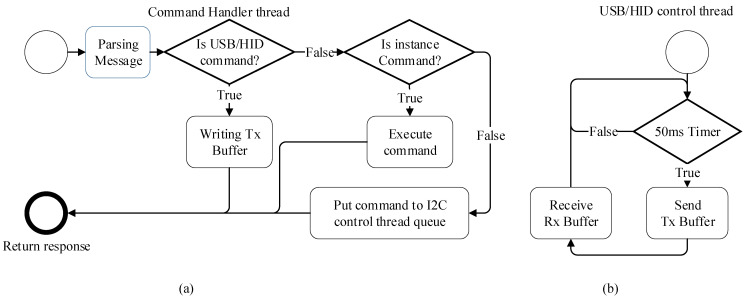
Real-time PCR controller flowchart: (**a**) command handler thread and (**b**) USB/HID control thread.

As illustrated in Figure 8, the command handler thread processes HTTP requests by distinguishing between MCU-related commands and instance commands. The instance commands are executed immediately within the handler, while queued commands are forwarded to the USB/HID control thread for transmission to the MCU. This separation allows for asynchronous and non-blocking execution, improving system responsiveness during PCR operation.

The internal firmware of the MCU executes time-sensitive tasks such as temperature regulation, sensor reading, and LED control every 2 ms as part of the PCR control task. Additionally, a USB communication task is scheduled every 30 ms to handle transmission and reception of 64-byte packets. These periodic tasks ensure precise thermal cycling and reliable fluorescence data collection. A detailed breakdown of this firmware process, including timer-based scheduling, is provided in Supplementary Figure S6.

### 3.4. Operational Procedure of the System

#### 3.4.1. Protocol Manager Operation

**Figure 9:**
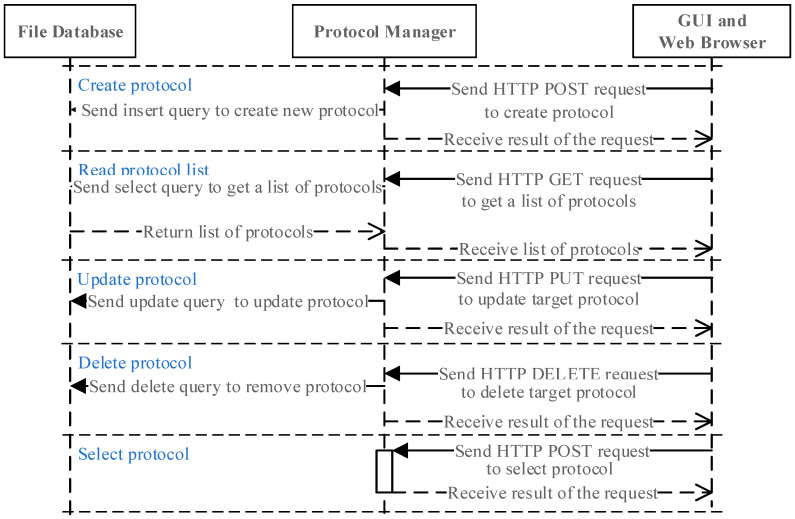
Sequence diagram of the protocol manager operation.

Figure 9 shows the sequence diagram of the protocol manager, which handles HTTP requests for protocol retrieval, storage, modification, and deletion. Protocol selection triggers a “Select protocol” request, generating a binary file (“recent protocol”), which is accessed by the task manager for execution.

#### 3.4.2. Protocol Execution and Stopping

**Figure 10:**
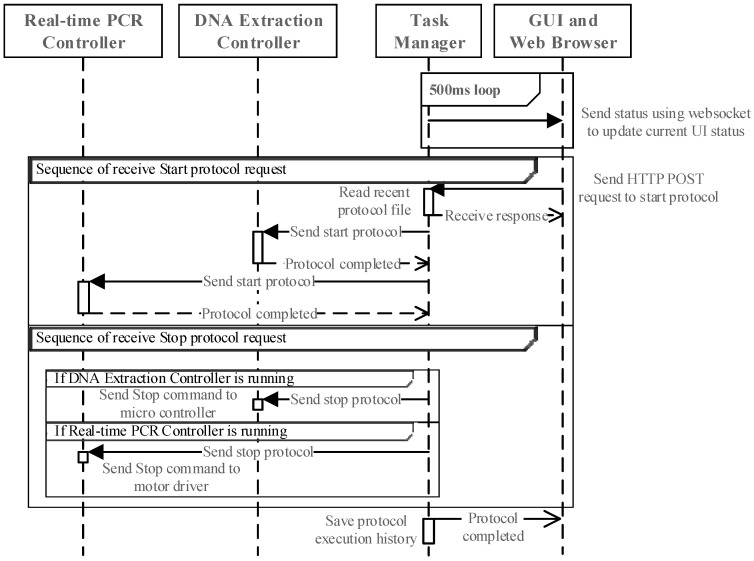
Execution and stopping sequence for molecular protocols.

Figure 10 outlines the process for initiating or terminating a protocol. The task manager updates the web-based GUI at 500 ms intervals via WebSocket. When execution begins, the task manager retrieves the DNA extraction protocol and sends it to the DNA extraction controller. Upon completion, the real-time PCR protocol is similarly executed. If a stop request is received, the appropriate controller is instructed to halt its operation. The final execution results are stored in the database for future reference.

#### 3.4.3. DNA Extraction Protocol Execution

**Figure 11:**
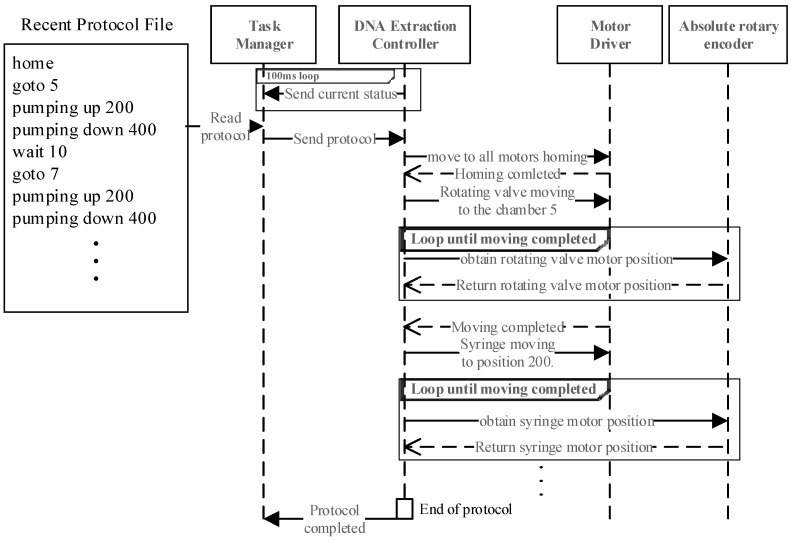
Execution sequence of a DNA extraction protocol.

Figure 11 represents an example execution of the DNA extraction protocol. The DNA extraction controller transmits status updates every 100 ms via WebSocket. Commands are executed sequentially, ensuring precise control using absolute rotary encoders. Once completed, the task manager transitions to the real-time PCR protocol.

#### 3.4.4. Real-Time PCR Protocol Execution

**Figure 12:**
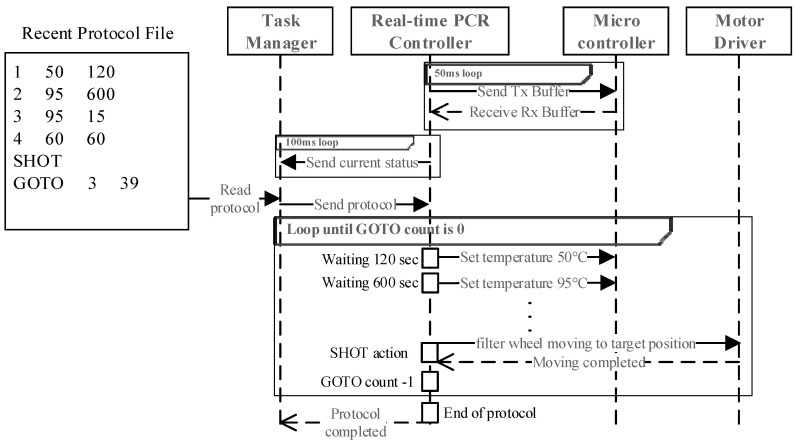
Execution sequence of a real-time PCR protocol.

Figure 12 illustrates the execution of a real-time PCR protocol following DNA extraction. The real-time PCR controller updates the task manager every 100 ms via WebSocket and communicates with the microcontroller at 50 ms intervals over USB/HID. Commands are executed sequentially, with looped actions handled via “GOTO” commands. Upon completion, results are stored for analysis. During the “SHOT” phase, the SBC triggers excitation LEDs and rotates the filter wheel to align the emission filter with the photodiode. Once aligned, it commands the MCU to acquire fluorescence intensity via ADC. This is repeated sequentially for each target dye.

### 3.5. Performance Evaluation

#### 3.5.1. Software Architecture Evaluation

To validate the proposed software architecture prior to full system integration, a hardware-based emulator—refined from our previous implementation [39]—was constructed using the same system firmware and a web-based GUI. This platform enabled real-time testing of control logic, module communication, and interface responsiveness under practical operating conditions (Figure S7). The system was integrated into a fully automated POC-MD device, currently undergoing clinical testing for simultaneous detection of *Chlamydia trachomatis* (CT) and *Neisseria gonorrhoeae* (NG). Four auxiliary tools were developed for system monitoring and troubleshooting:

(1) Extractor Controller Monitoring: Implemented using Vue.js, this web GUI includes command execution, register observation, and motor control functions.
(2) PCR Controller Monitoring: This tool, developed in Jupyter Notebook 6.5.4, verifies protocol execution and monitors temperature, fluorescence intensity, cycle count, and remaining time.
(3) Diagnostic History Viewer: A standalone GUI displaying diagnostic records, including patient data and test results, with options for review and report generation.
(4) Reference Fluorescence Unit (RFU) Navigator: A Python-based tool for browsing and visualizing PCR amplification curves, allowing multiplex fluorescence comparison.

These tools were independently developed without modifying the core system architecture, demonstrating the flexibility and scalability of the proposed system for clinical applications.

This revised implementation result demonstrated robustness, automation capabilities, and adaptability for clinical molecular diagnostics of the proposed software architecture.

#### 3.5.2. PCR Chip Verification with Standard DNA

To verify the performance of the PCR chip, amplification tests were conducted using a range of DNA concentrations. The reaction mixture consisted of 15 µL of master mix, 7.5 µL of PCR mix, 3 µL of distilled water, and 4.5 µL of DNA solution. The mixture was loaded into the cartridge’s PCR mixing chamber and subsequently transferred to the PCR chip for amplification.

**Figure 13:**
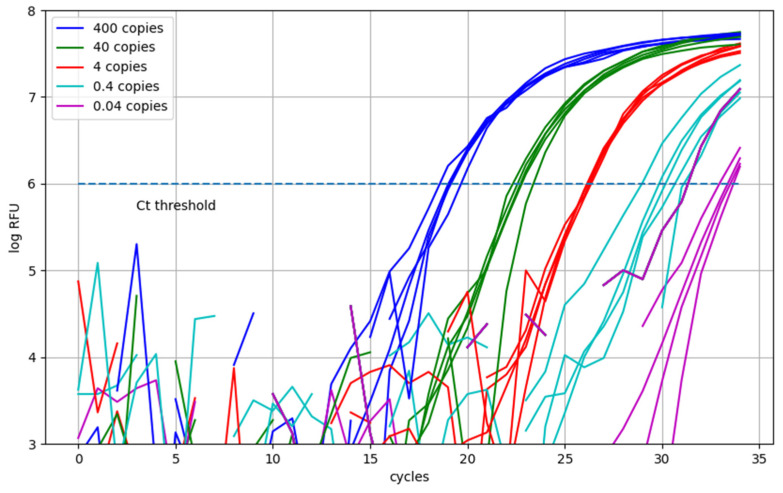
Real-time amplification curves for serially diluted template concentrations (400 to 0.04 copies per reaction). The dashed line represents the Ct threshold.

Figure 13 presents the real-time amplification curves obtained from five replicates at each of five template concentrations, ranging from 400 to 0.04 copies per 30 µL reaction. The dashed line indicates the log RFU threshold of 6, which was empirically determined as the optimal threshold minimizing the average Ct variability across all concentrations.

Table 1 summarizes the mean and standard deviation of the Ct values for each concentration. Notably, successful amplification was observed in all five replicates even at the lowest tested concentration (0.04 copies per 30 µL reaction), although the amplification curves at this concentration exhibited greater variability and delayed threshold crossing compared to higher concentrations.

In this preliminary evaluation, 0.04 copies per 30 µL reaction (~1.3 copies/mL) yielded consistent amplification in all five replicates with Ct values under 40 cycles. While this result suggests that 0.04 copies/reaction is a viable limit of detection, a definitive LoD determination according to Clinical and Laboratory Standards Institute (CLSI) guidelines (≥95% detection in ≥20 replicates) will be conducted during the clinical validation stage.

### 3.6. Clinical Performance Context and Prior Validation

Although the proposed system is based on a previously validated cartridge and PCR architecture, the IoMT-based implementation itself has not yet been clinically evaluated due to ongoing regulatory processes. However, a previous study by Lim et al. [24] evaluated the clinical performance of the earlier version of the LabGenius CT/NG platform (BIOMEDUX Co., Ltd., Suwon, Republic of Korea). The core cartridge structure and real-time PCR amplification design of this system are similar to those of the architecture presented in this study, including the closed-cartridge workflow and hardware-based fluorescence detection mechanism. As only the software architecture and IoMT interface have been modified, while the cartridge and hardware remain unchanged, the prior biochemical validation remains valid for the current system configuration. *Chlamydia trachomatis* and *Neisseria gonorrhoeae* were selected as the target pathogens based on their global prevalence, high asymptomatic transmission rates, and their clinical importance in early detection and control through point-of-care diagnostics.

That system shares the core cartridge structure and real-time PCR amplification design with the architecture presented here, including the closed-cartridge workflow and hardware-based fluorescence detection mechanism. As only the software architecture and IoMT interface have been modified, while the cartridge and hardware remain unchanged, the prior biochemical validation remains valid for the current system configuration.

In the referenced study [24], a total of 510 clinical specimens (343 vaginal swabs and 167 urine samples) were tested using the LabGenius C-CT/NG-BMX assay (BIOMEDUX Co., Ltd., Suwon, Republic of Korea) and compared against a commercial system, BD MAX CT/GC/TV (Becton Dickinson, Franklin Lakes, NJ, USA). The overall percent agreement (OPA) with BD MAX was 89.2–93.3% for *Chlamydia trachomatis* and 97.7–98.8% for *Neisseria gonorrhoeae*, with Cohen’s kappa coefficients indicating strong agreement (0.78–0.91 for CT, 0.92–0.95 for NG). The complete diagnostic cycle—from sample lysis to result reporting—takes approximately 60 min, including DNA extraction and real-time PCR amplification.

However, when compared with consensus results based on multiple PCR assays, the OPA for NG dropped to 50.0%, suggesting that further optimization or test standardization may be required for certain specimen types or use cases.

The limit of detection (LoD_95_) in the prior study was 9.9 and 7.8 copies/μL for CT and 7.6 and 5.1 copies/μL for NG in vaginal swabs and urine samples, respectively. Additionally, precision testing showed within-laboratory variation of less than 1.5% for Ct values, and no cross-reactivity was observed with non-target organisms or interferents.

These results, although obtained using a previous-generation system, support the feasibility of applying the proposed next-generation architecture in clinical environments, particularly for decentralized point-of-care molecular diagnostics.

## 4. Discussion

This study introduced a novel software architecture for a fully automated molecular diagnostic system, designed to improve operational efficiency and ease of use. Unlike traditional hierarchical software architecture, the proposed system adopted a distributed structure, dividing functionality between DNA extraction and real-time PCR processes. By using independent software modules that communicated via HTTP-based APIs, the system allowed for greater flexibility and modularity, enabling easier maintenance and upgrades. The integration of a web-based GUI enabled system access through a standard web browser without requiring specialized software. The adoption of a reactive framework allows clear separation between UX design and core system logic, thereby supporting future collaboration between software developers and interface designers during iterative refinement.

One of the key advantages of this system was its reliance on low-cost, open-source hardware components. The Raspberry Pi main controller, along with the PIC18F4553 microcontroller and L6470 motor controllers, provided a cost-effective alternative to commercial diagnostic systems while maintaining high performance. Furthermore, the software was developed in Python, a widely used and easy-to-learn programming language, making the system highly adaptable to future modifications and expansions. The adoption of WebSockets-based real-time communication significantly reduced latency, improving response times during protocol execution. Additionally, the system enhanced processing efficiency and minimized errors by optimizing microfluidic cartridge operations. The inclusion of a hardware-based emulator for pre-deployment validation ensured that potential issues were identified and resolved before implementation, further increasing reliability. The system’s architecture fulfills two core REASSURED criteria—real-time connectivity and ease of specimen collection—reinforcing its suitability for decentralized diagnostic applications and field deployment.

This interpretation is consistent with the recent literature emphasizing the importance of real-time connectivity and automation in IoMT-based molecular diagnostics [1,2]. The WebSocket-based control and feedback architecture implemented here enables low-latency interaction between the GUI and embedded controllers, enhancing operator awareness and workflow efficiency. Additionally, the system’s compatibility with non-invasive sample types and simplified cartridge operation supports the broader trend toward “sample-to-answer” diagnostic solutions that minimize user burden while maintaining analytical sensitivity [15]. Moreover, the modular software architecture enables remote configuration and seamless protocol updates, allowing the system to evolve in response to clinical feedback or changing operational requirements in decentralized healthcare environments. Such remote configurability becomes especially valuable when managing a fleet of diagnostic devices deployed across multiple decentralized sites. Although the system is designed for IoMT-based connectivity, all core diagnostic functions are executed locally on the SBC. In the event of network instability or temporary cloud service interruptions, diagnostic processes continue uninterrupted, and data are cached locally for upload upon reconnection.

In a prior clinical study using an earlier generation of the proposed system, a total of 510 genitourinary specimens were tested using a self-contained cartridge and a real-time PCR architecture similar to that presented in this study [41]. The results demonstrated high concordance with a commercially available PCR platform, with detection limits below 10 copies/μL and strong agreement in both CT and NG detection. While the current version has not yet undergone clinical testing, these findings provide indirect validation for the system’s design and highlight its clinical applicability in point-of-care diagnostic settings.

To further contextualize the proposed system, a comparison with recent IoMT-integrated nucleic acid amplification platforms is summarized in Table 2. While previous studies [42,43,44] have demonstrated portable diagnostic devices with IoMT features, most lack real-time bi-directional control, protocol-level reconfigurability, or sample preparation automation. Among these devices, the Raspberry Pi-based architecture proposed in [44] possesses a compatible hardware foundation but implements only partial monitoring via a web dashboard. In contrast, our system leverages WebSocket-based architecture to enable comprehensive device interaction and control, including emulator-driven protocol testing and automated cartridge workflows.

The proposed system can be utilized for a broad range of applications beyond those presented in this study. In clinical diagnostics, it can be deployed in hospitals, small clinics, and field laboratories to provide rapid, decentralized disease detection. The previous standalone system (LabGenius) is currently undergoing clinical testing at a public health center in Republic of Korea, with regulatory approval targeted for Q3 2025. The IoMT-based system proposed in this study comprises the same cartridge and detection architecture as the LabGenius system and is scheduled for clinical validation in Q4 2025, and its IRB approval is currently pending.

Its real-time monitoring capabilities also make it suitable for epidemiological surveillance, allowing for early outbreak detection and response. Additionally, the system’s integration with web-based technologies opens possibilities for remote healthcare applications, enabling decentralized testing and analysis through telemedicine platforms. The system can be further improved by integrating artificial intelligence algorithms for automated result interpretation, reducing the need for manual analysis. Miniaturization of the device could enhance its portability, making it more accessible for point-of-care testing. Furthermore, expanding its diagnostic capabilities to include additional disease targets would increase its applicability in diverse healthcare settings.

At present, specific AI models have not yet been implemented; the proposed IoMT system facilitates collection of comprehensive RFU curves and thermal cycling profiles. These data may support future development of more robust result classification algorithms beyond conventional cycle threshold–based methods.

In addition to swab and urine specimens, ongoing feasibility studies have demonstrated compatibility with blood and sweat samples using customized extraction protocols. The modular cartridge design and programmable workflow enable straightforward adaptation to additional sample types.

The IoMT-based system proposed in this study comprises the same cartridge and detection architecture as the LabGenius system and is scheduled for clinical validation in Q4 2025, and its IRB approval is currently pending. The current limit-of-detection (LoD) assessment was based on five replicates, and although results were consistent, the evaluation does not meet statistical criteria for formal LoD confirmation. Similarly, device-to-device reproducibility has not yet been established, as only a single prototype was tested. These limitations will be addressed in the clinical validation and scale-up phases, which will include ≥20 replicate tests for LoD determination and inter-device performance comparisons under standardized conditions.

As part of this process, data privacy and cybersecurity considerations—such as TLS-encrypted communication, token-based access control, and compliance with healthcare regulations—will be addressed during productization and large-scale deployment. In the current prototype, each functional module (e.g., extraction and amplification) operates as an independent FastAPI-based WebSocket server, and the system controller exposes REST APIs via a FastAPI HTTP server. This architecture enables modular development and real-time coordination within a local network. However, secure deployment will require full integration of security protocols across all communication channels. While modern cloud platforms provide encryption, authentication, and logging primitives, these features must be appropriately configured and integrated by the development team to meet end-to-end security requirements in accordance with HIPAA, GDPR, or equivalent standards.

## 5. Conclusions

This study presents an advanced cloud-based software framework for fully automated molecular diagnostics, building upon previous research to address key limitations related to latency, microfluidic efficiency, and user interaction. The proposed system successfully integrated low-cost hardware components with a distributed software architecture, enhancing both flexibility and reliability. Experimental validation confirmed its effectiveness, demonstrating its feasibility for deployment in medical and remote diagnostic settings. By leveraging modern web technologies and real-time communication protocols, the system offers a robust and scalable solution for point-of-care molecular diagnostics. While clinical testing of this system is forthcoming, it is based on a previously validated cartridge and PCR architecture that demonstrated strong agreement with commercial diagnostic platforms in a prior study involving over 500 patient specimens. These results support the clinical potential of the proposed design for decentralized molecular testing.

## Figures and Tables

**Table 1:** Mean and standard deviation of Ct values across replicates for each template concentration (values are expressed as mean ± standard deviation).

Copies/Reaction	400	40	4	0.4	0.04
Mean ± std.	19.1 ± 0.35	22.8 ± 0.35	26.2 ± 0.08	30.2 ± 0.74	33.0 ± 0.85

**Table 2:** Comparison of proposed system with related IoMT-enabled nucleic acid amplification platforms.

Feature	[42]	[43]	[44]	This Study (2025)
Amplification method	qPCR (MEMS)	LAMP (colorimetric)	RT-LAMP (fluorescence)	Real-time PCR (qPCR) (fluorescence)
IoMT communication	Bluetooth + LTE	Wi-Fi (image upload)	Web dashboard	WebSocket (bi-directional)
Sample preparation	No	No	No	Fully automated
Real-time remote control	No	No	Partial (monitoring only)	Yes
Remote protocol update	No	No	Limited	Yes
Web-based GUI	Basic app	Web image viewer	Dashboard only	Full control and monitoring
Emulator-driven SW validation	No	No	No	Yes
Platform	unknown	ESP32	Raspberry Pi 4	Raspberry Pi 3 A+

## Data Availability

The data presented in this study are included in the article and its Supplementary Materials. Further inquiries can be directed to the corresponding author.

## Supplementary Materials

The following supporting information can be downloaded at: https://www.mdpi.com/article/10.3390/s25144426/s1, Figure S1: LabGenius™ cartridge overview; Figure S2: System-level schematic of the previous platform; Figure S3: Photograph of the proposed diagnostic system; Figure S4: GUI screenshot of the proposed diagnostic system; Figure S5: Thread structure of the DNA extraction controller; Figure S6: PCR controller firmware task scheduling; Figure S7: Emulator hardware photographs.
