# Supplementary material for: IoMT Architecture for Fully Automated Point-of-Care Molecular Diagnostic Device
Source: Sensors (Basel). 2025 Jul 16;25(14):4426. doi: 10.3390/s25144426 (PMC12300902; doi:10.3390/s25144426)
Supplement: Supplementary file 1 [file sensors-25-04426-s001.zip › sensors-3692779-supplementary.pdf]

Supplementary Materials for

IoMT Architecture for Fully Automated Point-of-Care Molecular Diagnostic Device

Authors: MinGin Kim, Byeong Heon Kil, Mun Ho Ryu, and Jong Dae Kim

Figure S1.

Overview of the LabGenius™ cartridge used in both the previous and current systems. (a) Side view showing the integration of the extraction body and PCR chip. (b) Top view of the extraction body with example reagent allocations. (c) Cross-sectional schematic of the cartridge structure. (d) Rear view of the PCR chip illustrating the integrated heater pattern and thermistor for thermal cycling control. (Adapted from Kil et al., Sensors 2021, 21, 6980.)

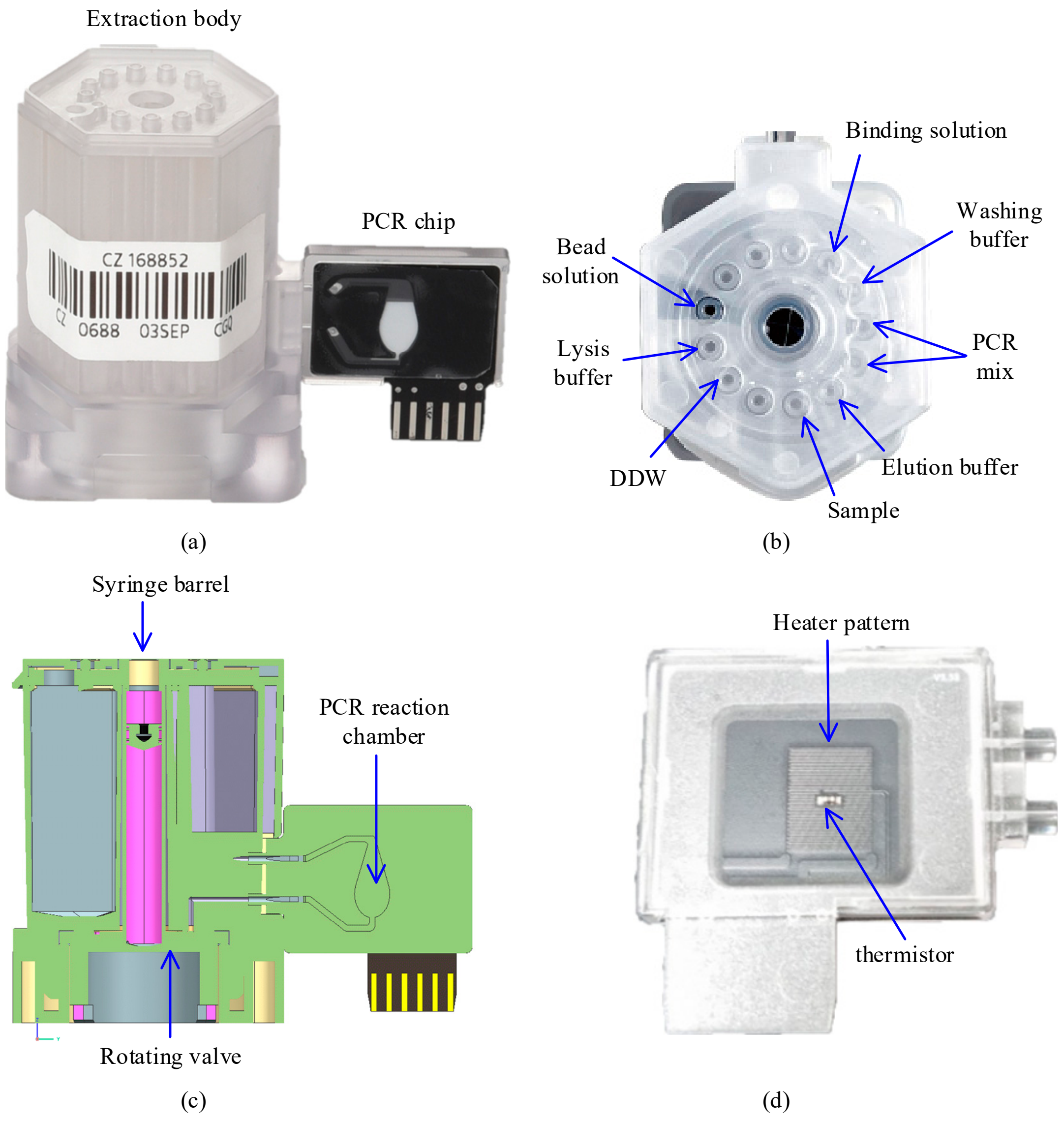

Figure S2.

System-level block diagram of the previously reported molecular diagnostic platform architecture. The schematic shows the overall integration of the SBC, microcontroller, motion control modules, and sensor-actuator interfaces necessary for automated DNA extraction, amplification, and fluorescence detection. (Adapted from Kil et al., Sensors 2021, 21, 6980.)

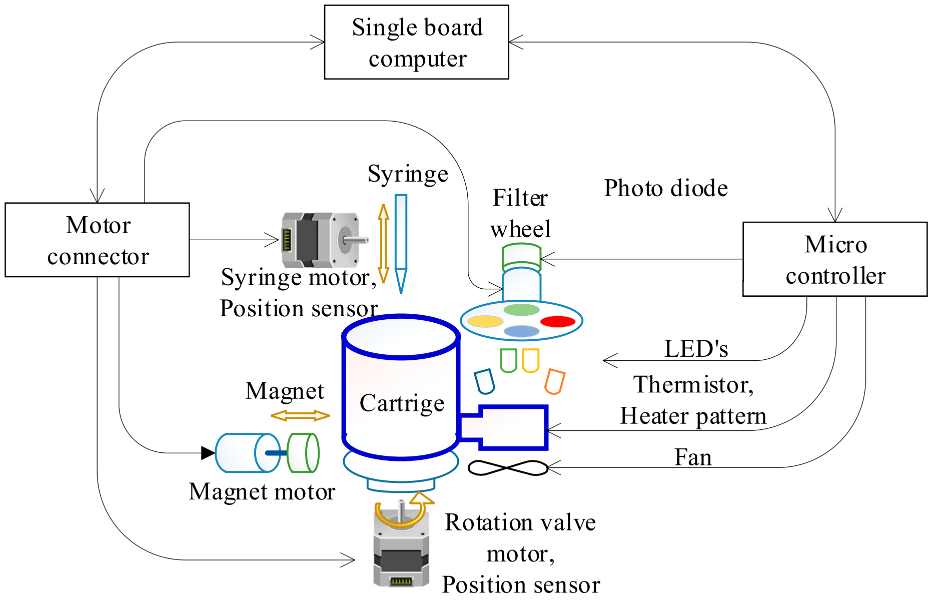

Figure S3.

Photograph of the implemented diagnostic system. Key components—including the rotation stage, syringe actuator, magnetic separation unit, and optical detection module—are labeled to illustrate the physical layout of the integrated hardware. The optical module shows the motor housing for the filter wheel; however, the filter wheel itself and the internal LED array are enclosed and not visible in this view.

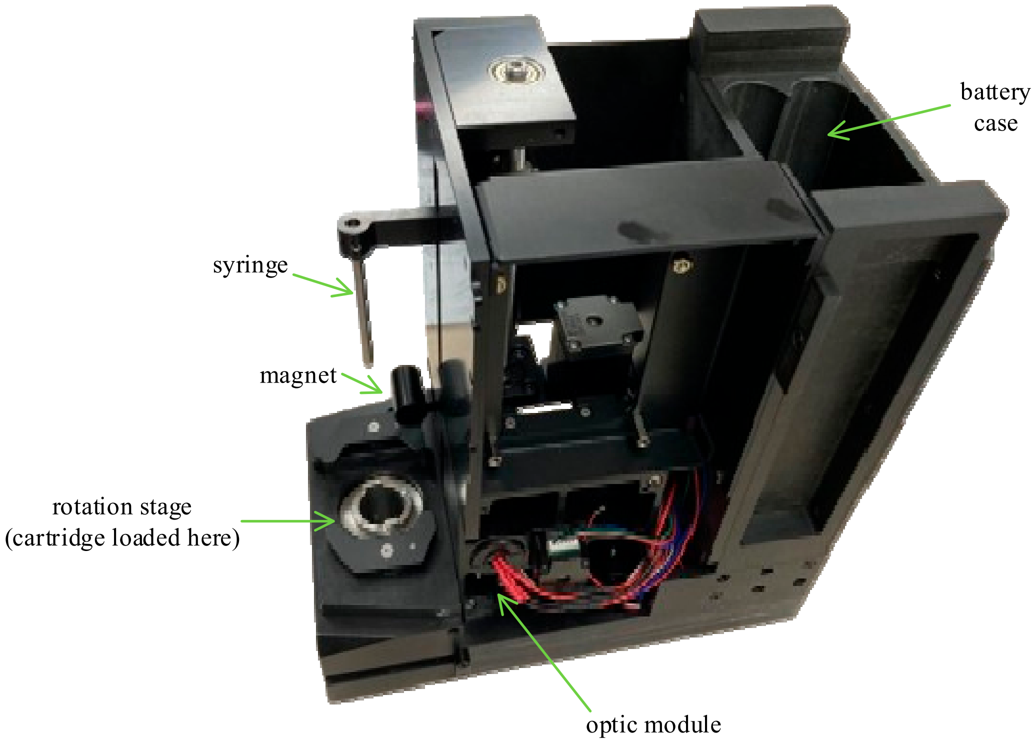

Figure S4.

Screenshot of the main page of the web-based graphical user interface (GUI) used in the proposed diagnostic system. The interface was developed using the React library and Bootstrap framework and consists of three pages: a main page for monitoring, a setup page for protocol configuration, and an editor page for protocol editing.

The main page includes six component groups and one real-time amplification plot. The Connection group displays the serial number of the connected device and its connection status. The Progress group shows the selected protocol, the current operational state, and the remaining PCR time. The Device group displays the current temperature of the PCR chip.

The Plot area visualizes real-time fluorescence data, with the x-axis representing PCR cycles and the y-axis showing sensor values obtained from the PCR controller. The CT Value group allows selection of fluorescence channels via image buttons and displays the corresponding Ct values upon completion. The Protocol group contains dropdowns for selecting stored protocols, buttons to start/stop the system, and a navigation button to the setup page. Lastly, the Result group shows a table listing the detection results (positive or negative) for each fluorescence target (Adapted with minor modifications from Kil et al., Sensors 2021, 21, 6980.).

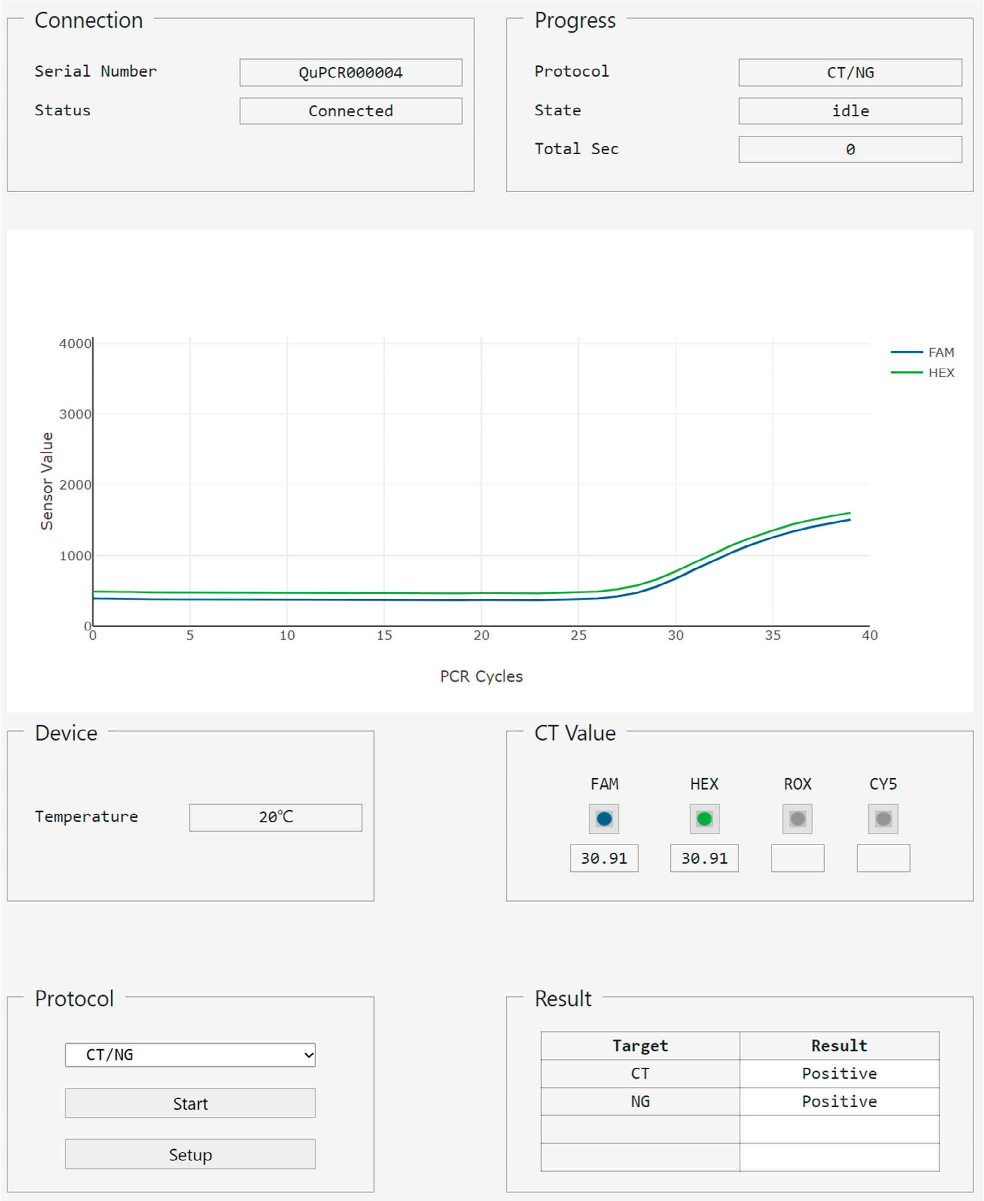

Figure S5.

Detailed thread structure of the DNA extraction controller.

The figure presents the internal software threading model and command parsing flow. The command handler thread separates instance commands from queued commands, and the I2C and GPIO threads manage low-level actuator control.

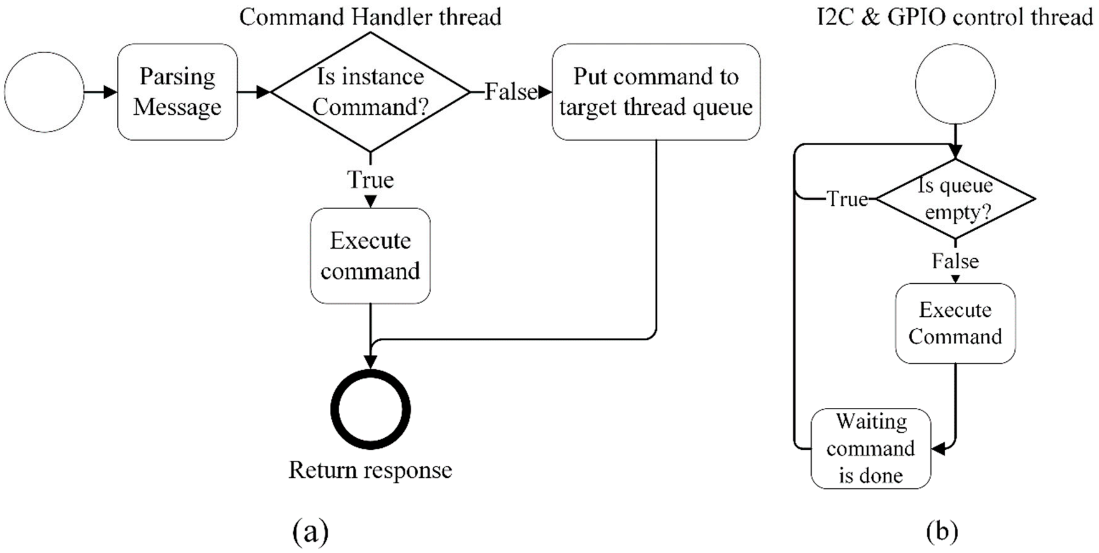

Figure S6.

Firmware task scheduling structure of the PCR controller.
This figure shows the MCU firmware architecture with 2ms and 30ms timers used for temperature control, USB communication, and fluorescence signal handling.

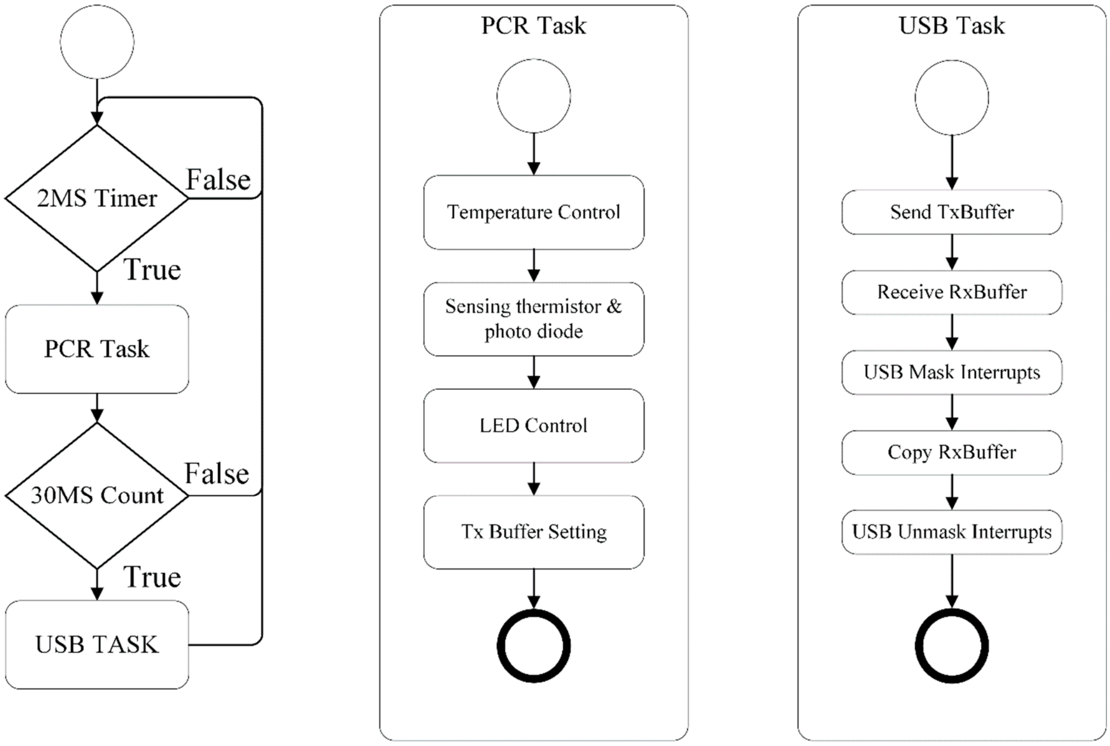

Figure S7.

Photographs of the hardware-based emulator platform used for validating the proposed software architecture.
(a) Top view of the emulator system. The SBC module (Raspberry Pi 3A+), which is hidden beneath the motor controller board, serves as the main controller and is connected to a stepper motor and a Hall effect sensor via the

motor controller board. A 12 V to 5 V DC converter supplies power to both the controller and peripherals. The rotation valve, syringe, and filter wheel stepper motors are functionally emulated using a single stepper motor. A servo motor for the extraction magnet is also included for magnet positioning. The microcontroller was replaced with a PIC18F2553 (ICP12-USB stick), which differs from the PIC18F4553 used in the main system only in the number of GPIO pins. ADC inputs for temperature and fluorescence were bypassed using virtual values.
(b) Rear view of the motion controller board. The board includes five SC18IS602B chips for I^2C-to-SPI bridging, three L6470 stepper motor drivers, and encoder connectors.

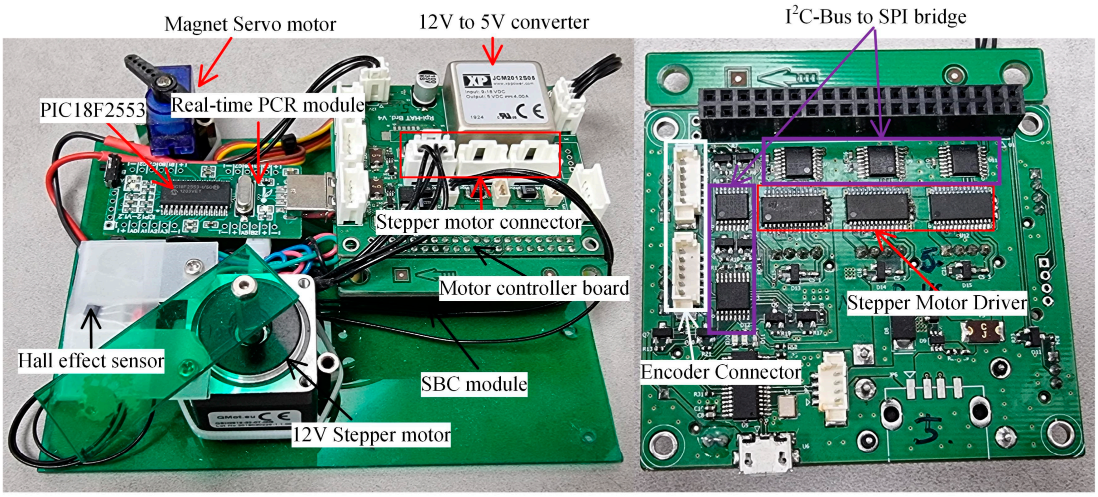

(a) (b)

- Figures S1, S2, and S4 are adapted with minor modifications from our previous publication: Kil et al., Sensors 2021, 21, 6980.
